# Colonic diverticular perforation by a migrated biliary stent: A case report with literature review

**DOI:** 10.1097/MD.0000000000028392

**Published:** 2021-12-30

**Authors:** Tae Young Park, Sung Woo Hong, Hyoung-Chul Oh, Jae Hyuk Do

**Affiliations:** a Division of Gastroenterology, Chung-Ang University College of Medicine, Seoul, Republic of Korea; b Department of General Surgery, Inje University Seoul Paik Hospital, Seoul, Republic of Korea.

**Keywords:** biliary stent, bowel perforation, endoscopic retrograde, endoscopic retrograde cholangiopancreatography

## Abstract

**Rationale:**

Plastic endobiliary stents, after endoscopic retrograde cholangiopancreatography, can get spontaneously dislocated from the common bile duct and migrate intothe distal bowel. Most migrated biliary stents are removed with the passing of stool. However, migrated biliary stents can cause bowel perforation, albeit rarely, and surgical intervention may be required. Recently, we observed a colonic diverticular perforation caused by a migrated biliary stent, and we have reported this case with a review of the literature.

**Patients concerns:**

A 74-year-old man presented with severe right lower quadrant pain after biliary stent insertion 1month ago.

**Diagnoses:**

Abdominal computed tomography revealed perforation of the proximal ascending colon by the migrated biliary stent, combined with localized peritonitis.

**Interventions:**

Emergency diagnostic laparoscopic examination revealed penetration of the proximal ascending colon by the plastic biliary stent, and right hemicolectomy was performed.

**Outcomes:**

On pathological examination, colonic diverticular perforation by the biliary stent was confirmed. The patient was discharged without any additional complications.

**Lessons:**

Endoscopic retrograde cholangiopancreatography endoscopists must always be cautious of the possibility of stent migration in patients with biliary stents in situ. In cases of biliary stent dislocation from the common bile duct in asymptomatic patients, follow-up with serial, plain abdominal radiographs, and physical examination is needed until confirmation of spontaneous passage through stool. In symptomatic cases suggesting peritonitis, abdominal computed tomography scan confirmation is needed, and early intervention should be considered.

## 1 Introduction

Endoscopic biliary stents have been widely used for internal biliary drainage during endoscopic retrograde cholangiopancreatography (ERCP). Dislocation and migration of the endobiliary stent from the common bile duct (CBD) occasionally occurs.[^1^,^2^] Dislocated biliary stents usually migrate to the distal bowel owing to peristalsis, and spontaneously pass out with feces, not requiring additional intervention so long as it does not cause symptoms.^[3]^ However, distal bowel perforation by migrated biliary stent occurs rarely, and it may require surgical intervention.[^3^,^4^,^5^,^6^,^7^] Herein, we report a case of colonic diverticular perforation caused by a migrated biliary stent, which is a very rare, late complication of ERCP, with a comprehensive review of previously reported cases.

## 2 Case report

**Figure 1:**
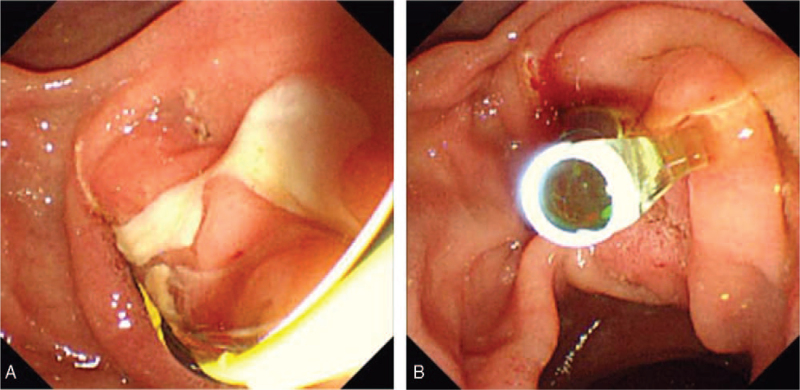
(A) Large amount of pus drained through the papilla. (B) After removal of the common bile duct (CBD) stone, a straight type plastic biliary stent inserted into the CBD to control acute suppurative cholangitis.

**Figure 2:**
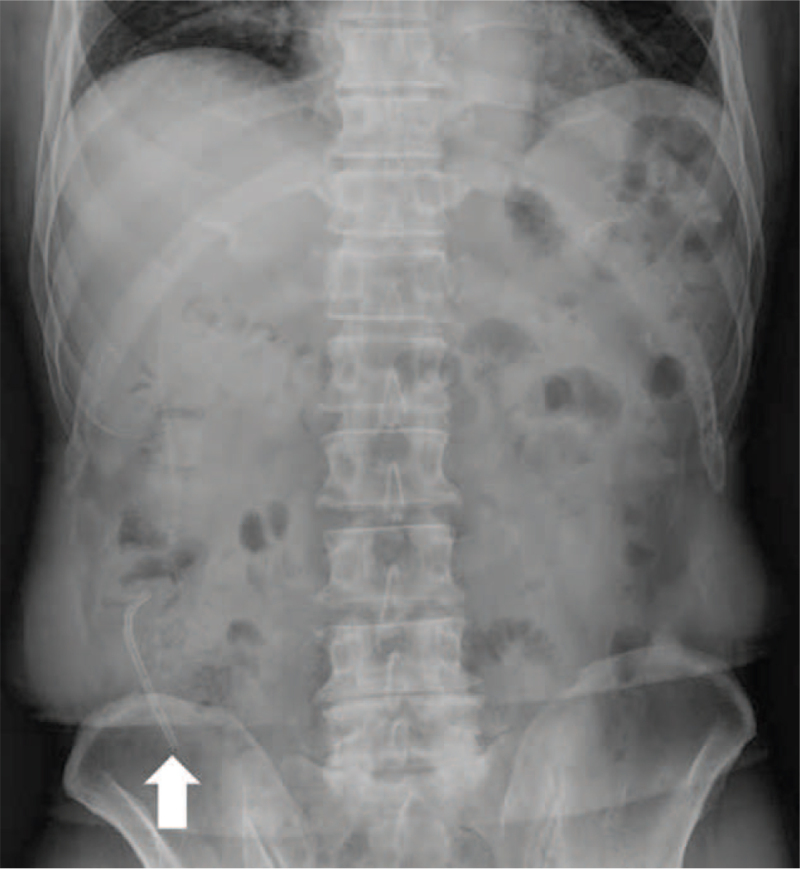
On abdominal radiography, the migrated endobiliary stent (arrow) is noted in the right lower quadrant area.

**Figure 3:**
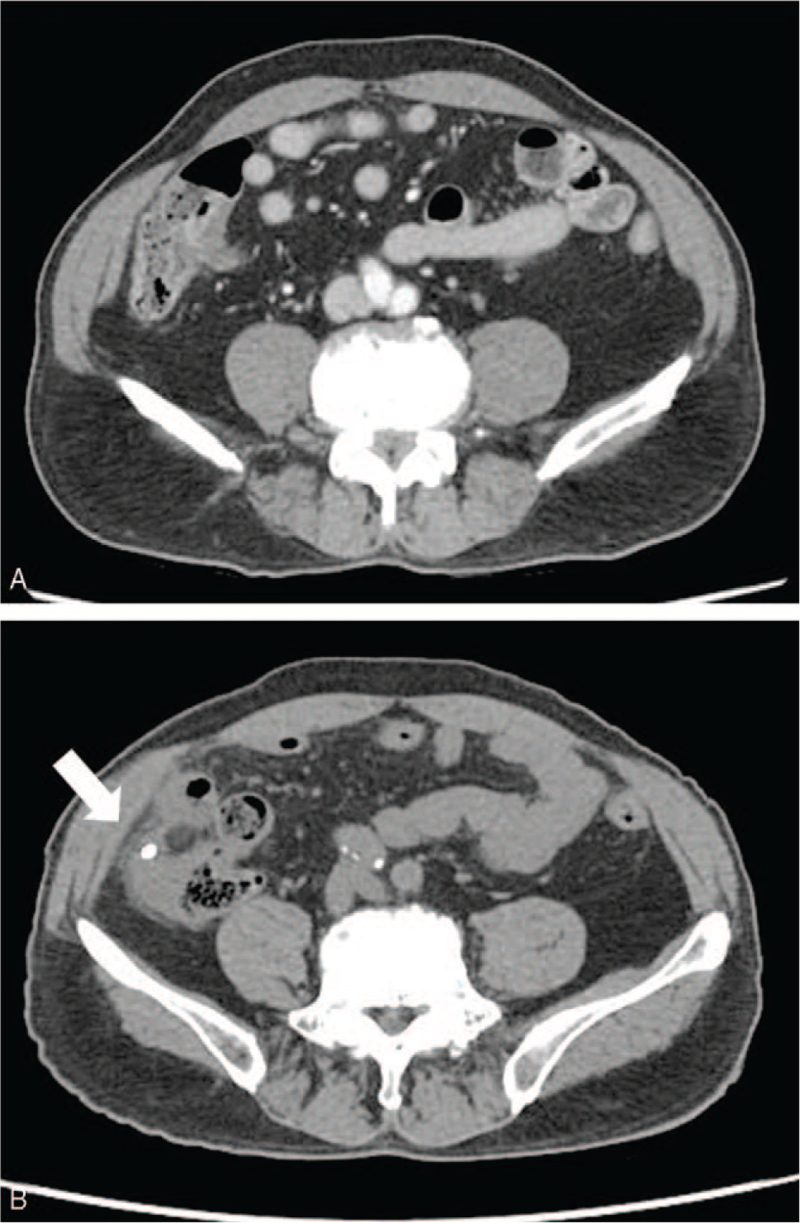
Abdominal computed tomography (CT) shows perforation of the proximal ascending colon with localized peritonitis by a migrated biliary stent.

**Figure 4:**
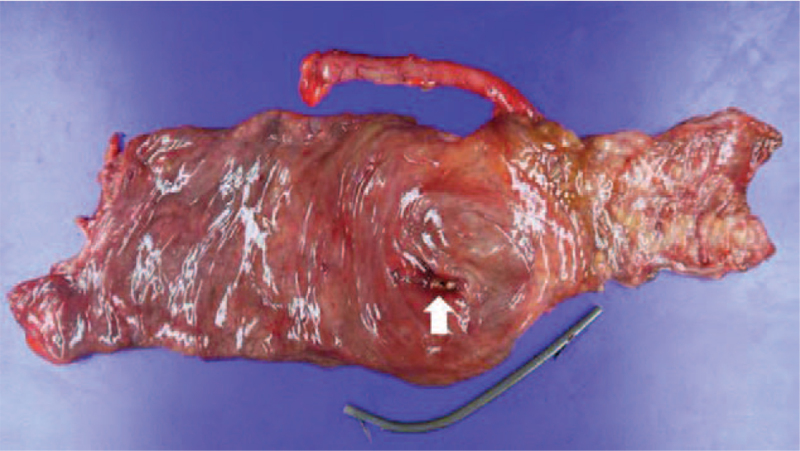
Pathological examination confirms diverticular perforation (arrow) in the proximal ascending colon associated with the endobiliary stent.

A 74-year-old man presented with abdominal pain. He had a medical history of ERCP and laparoscopic cholecystectomy due to cholangitis with CBD stones and cholecystitis with gallbladder stones about 1 year ago. Biliary colic, associated with fever and chills, was observed. Physical examination revealed the presence of tenderness (and the absence of rebound tenderness) in the right upper quadrant area. Icteric sclera was also observed. Laboratory findings revealed white blood cell counts of 10,730/mm^3^, hemoglobin levels of 15.3 g/dL, total bilirubin levels of 3.0 mg/dL, aspartate aminotransferase levels of 346 IU/L, alanine aminotransferase levels of 85 IU/L, alkaline phosphatase levels of 156 IU/L, and gamma-glutamyl transferase levels of 1010 IU/L. Abdominal computed tomography (CT) revealed multiple CBD stones with bile duct dilation. ERCP was performed to remove the CBD stones, followed by endoscopic retrograde biliary drainage with a 10 Fr x 7 cm straight-type plastic stent inserted into the CBD to control acute suppurative cholangitis (Fig. 1). The patient was discharged without early complications. One month later, he presented with severe right lower quadrant (RLQ) pain. Physical examination revealed tenderness in the RLQ area with rebound tenderness. Laboratory findings revealed white blood cell counts of 8700/mm^3^, hemoglobin levels of 14.5 g/dL, total bilirubin levels of 1.1 mg/dL, aspartate aminotransferase levels of 19 IU/L, alanine aminotransferase levels of 11 IU/L, alkaline phosphatase levels of 53 IU/L, gamma-glutamyl transferase levels of 99 IU/L, and C-reactive protein levels of 12.5 mg/dL. On plain abdominal radiography, the migrated biliary stent was found in the distal bowel (located in the RLQ area) (Fig. 2). Abdominal CT revealed perforation of the proximal ascending colon by the migrated biliary stent, combined with localized peritonitis. However, there was no evidence of ascites, pneumoperitoneum, or peritonitis (Fig. 3). Emergency diagnostic laparoscopic examination revealed penetration of the proximal ascending colon by the plastic biliary stent, and right hemicolectomy was performed. On pathological examination, colonic diverticular perforation by the biliary stent was confirmed (Fig. 4). The patient was discharged without any additional complications. The patient has provided informed consent for publication of the case. The study was approved by the Institutional Review Board of the Inje University Seoul Paik Hospital (IRB No. PAIK 2021-06-012-001).

## 3 Discussion

Biliary stent migration can occur in 5% to 10% of patients with endoscopic biliary stenting.^[1]^ The risk factor for biliary stent migration from the CBD to the distal bowel has not yet been established. In a retrospective cohort study, biliary plastic stent migration occurred more frequently in benign biliary strictures than in malignant biliary strictures.^[2]^ Distal migration was associated with long stents, and proximal and postcholecystectomy strictures, whereas proximal migration was associated with short stents, and distal and non-postcholecystectomy strictures.

Migrated plastic biliary stents in the large intestine, which have passed through the narrow diameter of the small intestine, rarely cause symptoms. Colon perforations due to migrated plastic biliary stents are very rare. The sigmoid colon was the most commonly involved segment.^[5]^ Bowel perforation by a dislocated endobiliary stent was associated with structural bowel abnormalities or variations, such as postoperative bowel adhesion, diverticulosis, hernia, or stricture.^[3]^

The detailed clinical features of the colon perforation cases by distal migrated biliary stents are summarized in Table 1. A total of 30 cases of colon perforation, including the current case, were identified. Most cases were associated with colonic diverticulum (20 out of 30 cases), and the most commonly involved colonic segment was the sigmoid colon (25 sigmoid colon, 1 cecum, 1 ascending colon, 1 splenic flexure, 1 rectum, 1 appendix). A total of 22 cases required surgical treatment, and 8 patients recovered by medical treatment without surgery.

**Table 1:** Clinical features of colon perforation by migrated plastic biliary stent.

Study	Age/sex	Risk factor	Indication for ERCP	Type of biliary stent	Time to migration	Location of perforation	Treatment
D’Costa 1994^[8]^	M/73	N/A	CBD cancer	N/A	N/A	Sigmoid	Surgery
Baty 1996^[9]^	F/86	Diverticulosis	Pancreas head cancer with CBD invasion	N/A	N/A	Sigmoid	Sigmoidectomy
Schaafsma 1996^[10]^	F/77	Diverticulosis	Acute cholangitis with CBD stone	Straight	6 mo	Sigmoid	Surgery
Lenzo 1998^[11]^	F/82	Diverticulosis	Acute cholangitis with CBD stone	Straight 10 Fr x 7.5 cm	4 wks	Sigmoid	Surgical primary closure
Størkson 2000^[12]^	M/86	N/A	Acute cholangitis with CBD stone	Straight 7 Fr x 5 cm	2 yrs	Sigmoid	Surgical primary closure
Figueiras 2001^[13]^	M/47	N/A	Chronic pancreatitis with distal biliary stricture	Straight 10 Fr x 10 cm	3 mo	Splenic flexure	Removal through colocutaneous fistula
Klein 2001^[14]^	F/70	Diverticulosis	CBD stone	Straight 7 Fr x 5 cm	3 yrs	Sigmoid	Surgery
Elliott 2003^[15]^	F/80	N/A	Acute cholangitis with CBD stone	Straight 10 Fr x 10 cm	4 mo	Sigmoid	Hartmann procedure
Diller 2003^[16]^	F/58	Diverticulosis	Post-LT bile duct stricture	Straight 7 Fr x 10 cm	1 mo	Sigmoid	Sigmoidectomy
Welhelm 2003^[3]^	F/85	Diverticulosis	CBD stone	Straight	N/A	Sigmoid	Sigmoidectomy
Anderson 2007^[17]^	F/80	Diverticulosis	CBD stone	Straight	5 mo	Sigmoid	Endoscopic removal
Namdar 2007^[7]^	F/65	N/A	Post-cholecystectomy bile leakage	Straight 12 Fr x 10 cm	3 mo	Rectum	Rectal resection
Bagul 2010^[18]^	F/79	Diverticulosis	Post-cholecystectomy bile duct stricture	Double pigtail 10 Fr x 9 cm	1 mo	Sigmoid	Endoscopic removal
Jafferbhoy 2011^[19]^	F/82	Diverticulosis	Post-cholecystectomy bile leakage	Straight 7 Fr x 7 cm	3 mo	Sigmoid	Endoscopic removal and clip closure
Lankisch 2011^[20]^	F/65	N/A	Pancreas head cancer with CBD invasion	Straight 10 Fr x 10 cm	2 wks	Sigmoid	Surgery
Malgras 2011^[21]^	73 y/o	Diverticulosis	Pancreas head cancer with CBD invasion	Straight 10 Fr x 5 cm	15 d	Sigmoid	Hartmann procedure
Wagemakers 2011^[22]^	F/76	Diverticulosis	CBD stone	N/A	1 mo	Sigmoid	Sigmoidectomy
Alcaide 2012^[23]^	M/73	Diverticulosis	CBD stone with benign biliary stricture	Straight 10 Fr x 12 cm	15 d	Sigmoid	Endoscopic removal and clip closure
Jones 2013^[24]^	M/66	N/A	Post-op CBD stricture	Straight	3 mo	Cecum	Endoscopic removal
Mady 2015^[25]^	M^∗^	Diverticulosis	Pancreas head cancer with CBD invasion	N/A	4 wks	Sigmoid	Hartmann procedure
Virgilio 2015^[5]^	Case 1, F^∗^ Case 2, F^∗^	DiverticulosisDiverticulosis	CBD stoneCBD stone	N/AStraight 12 Fr x 12 cm	N/AN/A	SigmoidSigmoid	Hartmann procedureEndoscopic removal
Chittleborough 2016^[26]^	M/73	Diverticulosis	Acute cholangitis with CBD stone	Straight 10 Fr x 5 cm	3 mo	Sigmoid	Hartmann procedure
Chou 2017^[27]^	F/85	N/A	CBD stone	N/A	N/A	Sigmoid	Endoscopic removal and clip closure
Siaperas 2017^[28]^	F/75	Diverticulosis	Post-op CBD stricture	Straight	1 mo	Sigmoid	Hartmann procedure with colostomy
Riccardi 2019^[29]^	F/79	Diverticulosis	CBD stone	Straight 10 Fr x 10 cm, Double pigtail 7 Fr	4 wks	Sigmoid	Hartmann procedure with colostomy
Marcos 2020^[6]^	F/65	Diverticulosis	CBD stone	Straight 10 Fr x 5 cm	1 yr	Sigmoid	Surgical primary closure
Pengermä 2021^[30]^	F/66	N/A	Chronic pancreatitis with distal biliary stricture	Straight, 10 Fr x 5 cm	4 d	Appendix	Appendectomy
Tao 2021^[31]^	M/54	N/A	Acute cholangitis with CBD stone, biliary pancreatitis	Straight	3 mo	Sigmoid	Sigmoidectomy+colostomy
Current case	M/74	Diverticulosis	Acute suppurative cholangitis with CBD stone	Straight, 10 Fr x 7 cm	1 mo	Proximal ascending	Rt. hemicolectomy

In conclusion, we report a case of perforation of the proximal ascending colon caused by a migrated biliary stent. ERCP endoscopists must always be cautious of the possibility of stent migration in patients with biliary stents in situ. In cases of biliary stent dislocation from the CBD in asymptomatic patients, follow-up with serial, plain abdominal radiographs and physical examination is needed until confirmation of spontaneous passage through stool. In symptomatic cases suggesting peritonitis, abdominal CT scan confirmation is needed, and early intervention should be considered.

## Author contributions

**Conceptualization:** Tae Young Park.

**Data curation:** Tae Young Park, Sung Woo Hong, Hyoung-Chul Oh.

**Methodology:** Sung Woo Hong, Hyoung-Chul Oh.

**Supervision:** Sung Woo Hong, Jae Hyuk Do.

**Validation:** Hyoung-Chul Oh.

**Writing – original draft:** Tae Young Park.

**Writing – review & editing:** Tae Young Park, Jae Hyuk Do.

## References

[1] Johanson JF Schmalz MJ Geenen JE. Incidence and risk factors for biliary and pancreatic stent migration. Gastrointest Endosc 1992;38:341–6. 1607087 10.1016/s0016-5107(92)70429-5

[2] Arhan M Odemiş B Parlak E. Migration of biliary plastic stents: experience of a tertiary center. Surg Endosc 2009;23:769–75. 18649099 10.1007/s00464-008-0067-x

[3] Wilhelm A Langer C Zoeller G. Complex colovesicular fistula: a severe complication caused by biliary stent migration. Gastrointest Endosc 2003;57:124–6. 12518151 10.1067/mge.2003.71

[4] Yaprak M Mesci A Colak T. Biliary stent migration with duodenal perforation. Eurasian J Med 2008;40:154–6. 25610053 PMC4261672

[5] Virgilio E Pascarella G Scandavini CM. Colonic perforations caused by migrated plastic biliary stents. Korean J Radiol 2015;16:444–5. 25741207 10.3348/kjr.2015.16.2.444 PMC4347281

[6] Marcos P Capelão G Atalaia-Martins C. Sigmoid perforation by a migrated plastic biliary stent. GE - Port J Gastroenterol 2020;27:215–8. 32509931 10.1159/000503076 PMC7250348

[7] Namdar T Raffel A-M Topp S-A. Complications and treatment of migrated biliary endoprostheses: a review of the literature. World J Gastroenterol 2007;13:5397–9. 17879415 10.3748/wjg.v13.i40.5397 PMC4171335

[8] D’Costa H Toy E Dennis MJ. Case report: intestinal perforation--an unusual complication of endoscopic biliary stenting. Br J Radiol 1994;67:1270–1. 7533042 10.1259/0007-1285-67-804-1270

[9] Baty V Denis B Bigard MA. Sigmoid diverticular perforation relating to the migration of a polyethylene endoprosthesis. Endoscopy 1996;28:781. 9007435 10.1055/s-2007-1005606

[10] Schaafsma RJ Spoelstra P Pakan J. Sigmoid perforation: a rare complication of a migrated biliary endoprosthesis. Endoscopy 1996;28:469–70. 8858249 10.1055/s-2007-1005523

[11] Lenzo NP Garas G. Biliary stent migration with colonic diverticular perforation. Gastrointest Endosc 1998;47:543–4. 9647386 10.1016/s0016-5107(98)70262-7

[12] Størkson RH Edwin B Reiertsen O. Gut perforation caused by biliary endoprosthesis. Endoscopy 2000;32:87–9. 10691280 10.1055/s-2000-87

[13] Figueiras RG Echart MO Figueiras AG. Colocutaneous fistula relating to the migration of a biliary stent. Eur J Gastroenterol Hepatol 2001;13:1251–3. 11711785 10.1097/00042737-200110000-00021

[14] Klein U Weiss F Wittkugel O. Migration of a biliary Tannenbaum stent with perforation of sigmoid diverticulum. ROFO 2001;173:1057. 11704920 10.1055/s-2001-18306

[15] Elliott M Boland S. Sigmoid colon perforation following a migrated biliary stent. ANZ J Surg 2003;73:669–70. 12887548 10.1046/j.1445-2197.2003.02698.x

[16] Diller R Senninger N Kautz G. Stent migration necessitating surgical intervention. Surg Endosc 2003;17:1803–7. 14508668 10.1007/s00464-002-9163-5

[17] Anderson EM Phillips-Hughes J Chapman R. Sigmoid colonic perforation and pelvic abscess complicating biliary stent migration. Abdom Imaging 2007;32:317–9. 16944034 10.1007/s00261-006-9067-2

[18] Bagul A Pollard C Dennison AR. A review of problems following insertion of biliary stents illustrated by an unusual complication. Ann R Coll Surg Engl 2010;92:W27–31. 10.1308/147870810X12659688852239 PMC5696895 20501006

[19] Jafferbhoy SF Scriven P Bannister J. Endoscopic management of migrated biliary stent causing sigmoid perforation. BMJ Case Rep 2011;2011: doi: 10.1136/bcr.04.2011.4078. 10.1136/bcr.04.2011.4078 PMC3089930 22696699

[20] Lankisch TO Alten TA Lehner F. Biliary stent migration with colonic perforation: a very rare complication and the lesson that should be learned from it. Gastrointest Endosc 2011;74:924–5. discussion 925. 21951480 10.1016/j.gie.2011.06.011

[21] Malgras B Pierret C Tourtier JP. Double sigmoid colon perforation due to migration of a biliary stent. J Visc Surg 2011;148:e397–9. 22075561 10.1016/j.jviscsurg.2011.09.011

[22] Wagemakers S Ibelings M. Colovesicular fistula after migration of a biliary stent. Ned Tijdschr Geneeskd 2011;155:A3615. 21914233

[23] Alcaide N Lorenzo-Pelayo S Herranz-Bachiller MT. Sigmoid perforation caused by a migrated biliary stent and closed with clips. Endoscopy 2012;44: (Suppl 2 UCTN): E274. 22814918 10.1055/s-0032-1309719

[24] Jones M George B Jameson J. Biliary stent migration causing perforation of the caecum and chronic abdominal pain. BMJ Case Rep 2013;2013:01–3. 10.1136/bcr-2013-009124 PMC3794329 24022897

[25] Mady RF Niaz OS Assal MM. Migrated biliary stent causing perforation of sigmoid colon and pelvic abscess. BMJ Case Rep 2015;2015:01–3. 10.1136/bcr-2014-206805 PMC4401935 25870211

[26] Chittleborough TJ Mgaieth S Kirkby B. Remove the migrated stent: sigmoid colon perforation from migrated biliary stent. ANZ J Surg 2016;86:947–8. 25078878 10.1111/ans.12796

[27] Chou ND Burbridge RA Jowell PS. Colonic perforation secondary to retained biliary stent. Am J Gastroenterol 2017;112:13. 10.1038/ajg.2016.414 28050041

[28] Siaperas P Ioannidis A Skarpas A. A rare cause for Hartmann's procedure due to biliary stent migration: a case report. Int J Surg Case Rep 2017;31:83–5. 28122319 10.1016/j.ijscr.2017.01.016 PMC5257179

[29] Riccardi M Deters K Jabbar F. Sigmoid diverticulitis and perforation secondary to biliary stent migration. Case Rep Surg 2019;2019:2549170. 31236301 10.1155/2019/2549170 PMC6545746

[30] Pengermä P Katunin J Turunen A. Appendiceal perforation due to biliary stent migration in a neutropenic patient with lung cancer receiving chemotherapy: a case report. Mol Clin Oncol 2021;15:136–9. 34055351 10.3892/mco.2021.2298 PMC8145604

[31] Tao Y Long J. Sigmoid colon perforation caused by migrated plastic biliary stents: a case report. Int J Colorectal Dis 2021;36:199–201. 32865713 10.1007/s00384-020-03728-2

